# Risk of dementia or Parkinson’s disease in the presence of Sjögren’s syndrome: A systematic review and meta-analysis

**DOI:** 10.3389/fnint.2022.1027044

**Published:** 2022-11-07

**Authors:** Zhen-Zhi Wang, Meng-Si Liu, Zhen Sun, Xu-Long Zhang, Mei-Ling Zhang, Kang Xiong, Feng Zhou

**Affiliations:** ^1^The First Clinical Medical College of Shaanxi University of Traditional Chinese Medicine, Xianyang, China; ^2^Department of Clinical Medicine, Hengyang Medical School, University of South China, Hengyang, China; ^3^Shaanxi Province Rehabilitation Hospital, Xi’an, China; ^4^The Affiliated Hospital of Shaanxi University of Traditional Chinese Medicine, Xianyang, China

**Keywords:** Sjögren’s syndrome, Parkinson’s disease, dementia, systematic review, meta-analysis, risk factor

## Abstract

**Objective:**

Evidence from observational studies suggests that Sjögren’s syndrome (SS) may contribute to an elevated risk of Parkinson’s disease (PD) and dementia. However, few studies have been undertaken to summarize and assess the consistency of the data quantitatively. Therefore, we evaluated the risk of dementia and PD in SS patients through a systematic review and meta-analysis approach.

**Methods:**

Two reviewers independently conducted a systematic search of PubMed, Embase, and Web of Science databases (updated to February 14, 2022) to identify published literature on the association between SS and dementia or PD. The risk estimates of dementia or PD in patients with SS were pooled using fixed or random-effects models.

**Results:**

Of the 631 studies initially searched, 10 were eventually included. Pooled results suggested that the risk of developing dementia significantly increased in patients with SS (HR = 1.24, 95% CI: 1.15–1.33, *P* < 0.001), and such risk in females with SS was similar to that in males. The risk of PD was 1.36 times higher in SS (HR = 1.36, 95% CI: 1.23–1.50, *P* < 0.001). The association between SS and PD risk appeared to occur primarily in female patients (female: HR = 1.28, 95% CI: 1.21–1.35; *P* < 0.001 vs. male: HR = 1.00, 95% CI: 0.87–1.16, *P* = 0.962, respectively). No significant effect of age was observed on the risk of developing PD and dementia in SS patients.

**Conclusion:**

Our study supports that people with SS are at higher risk of PD and dementia than the general population. Further studies are needed to elucidate the underlying mechanisms and to assess whether interventions for SS have the potential to affect dementia and PD development.

## Objective

Sjögren’s syndrome (SS) is an autoimmune disorder that plagues 35 million people worldwide. Its main clinical manifestations include dry eyes and dry mouth as well as lymphocytic infiltration of glandular tissues (Sjögren, 1933). SS is characterized by reduced salivary and lacrimal gland function, lymphocytic infiltration, elevated pro-inflammatory cytokines, and circulating autoantibodies. Seventy percent of patients experienced significant fatigue, which greatly disrupted their daily lives (Odani and Chiarini, 2019; Mæland et al., 2021). Among 30–50% of SS patients, extra-glandular symptoms involving the skin, joints, lungs, neurological system, and kidneys, as well as malignant lymphoma were observed (Fox, 2005; Malladi et al., 2012; Ramos-Casals et al., 2015). Recent studies have revealed that the prevalence of neurological manifestations of primary SS (pSS) ranges from 8 to 49% (Margaretten, 2017), such as cognitive dysfunction and dementia (Delalande et al., 2004; Blanc et al., 2013).

Since the beginning of the twenty first century, population aging has become a prominent issue, challenging all countries in the world (Beard et al., 2016). China now ranks first in the world regarding the number of older adults and the rate of aging (Wu et al., 2021). Dementia and PD have emerged as the most common neurodegenerative diseases threatening the health of the age (Jellinger, 2003), with high incidence, serious hazards, and irreversible neuronal damage to the brain (Hindle, 2010). The treatment of these diseases also places a heavy burden on families and society (Alzheimer’s Association, 2017; Kwon et al., 2017). This predicament is compounded by the dim prospect for the research and development of medication targeting such diseases.

An association between neuropsychiatric symptoms and autoimmune diseases has been increasingly noted. Several observational studies have explored the association between SS and dementia/PD. Blanc et al. (2013) reported that of 25 patients with SS, 15 suffered from cognitive impairment and 5 developed dementia, revealing the risk of dementia in patients with SS. Several large sample longitudinal cohort studies with long-term follow-up (9–15 years) also confirmed that baseline SS is significantly associated with elevated risks of PD and dementia (Wu et al., 2017; Liliang et al., 2018; Chen et al., 2019; Hsu et al., 2020). Although there is evidence of an association between SS and PD and dementia, no meta-analysis has been conducted to quantitatively summarize and examine data consistency for higher-quality evidence.

Therefore, the present study aimed to quantify the association between SS and dementia/PD. To achieve this end, data were collected to (1) investigate the comprehensive results of the association between SS and dementia, and that between SS and PD; (2) validate the main results by analyzing the results in different subgroups and determining the sources of heterogeneity; (3) test the robustness of the results through sensitivity analysis and assess the potential for publication bias.

## Methods

### Search strategy

The study was reported according to the Preferred Reporting Items for Systematic Review and Meta-analysis Protocols (PRISMA-p) statement (Moher et al., 2015). We systematically searched PubMed, Embase, and Web of Science for relevant literature on SS and dementia/PD from inception to February 14, 2022. The following keywords were used: “SS AND PD”, “SS AND Alzheimer’s disease OR dementia.” No restrictions were imposed on the publication type or language of the journal. The detailed search strategy is provided in Supplementary Table 1.

### Inclusion and exclusion criteria

Studies were eligible for inclusion if they were longitudinal case-control studies or cohort studies, entailed a clear definition of SS, dementia, and PD, or applied standard clinical diagnostic criteria to identify relevant cases. Included studies also should meet the following criteria: assessing the association between SS and dementia as well as SS and PD, or reporting effect estimates and corresponding 95% confidence intervals (CIs). The studies should also include relevant data to calculate hazard ratios (HRs) for the associations between SS and dementia/PD. We excluded reviews, conference abstracts, commentaries, reprinted literature, and studies with duplicated or incomplete data. To avoid omitting any studies, we manually searched the literature cited in the reference lists of included studies.

### Data extraction

The final screening results were compared by two authors (WZZ and SZ) based on the same inclusion and exclusion criteria, and any disagreements were resolved by third-party authors. An MS Excel form was created to record essential data from the included studies, including first author, year of publication, study type, country, sample size, the mean age of samples, exposure definition, factors adjusted for the outcome, eligible subgroups, and follow-up periods. All authors approved the final version of the template. Two independent reviewers (WZZ and SZ) extracted data using the pre-determined form, and any discrepancies were addressed through discussion. The corresponding authors of the included literature were also contacted for further information when necessary.

### Quality assessment

Two independent reviewers (WZZ and SZ) scored the quality of the included case-control and cohort studies, following the Newcastle-Ottawa Quality Assessment Scale. Studies were rated as high-quality if scored between 7 and 9, medium-quality (5∼6), and low-quality (≤4) (Stang, 2010).

### Statistical methods

The HRs were pooled to examine the relationship between SS and PD/dementia risk (Higgins et al., 2003). Heterogeneity was considered acceptable when *I*^2^ < 50% and *p* > 0.05, and a fixed-effects model was used for analysis. A random-effects model was applied when *I*^2^ ≥ 50% or *p* ≤ 0.05. HR, an effective indicator for meta-analysis, was used to calculate pooled effect values, and forest plots were drawn. Further subgroup analyses were conducted based on the available data from the included studies. Sensitivity analysis was performed to evaluate the stability of the results. Publication bias was assessed using Begg’s and Egger’s tests and graphing funnel plots. All statistical analyses were done using Stata 16.0 software.

## Results

### Study selection

**FIGURE 1:**
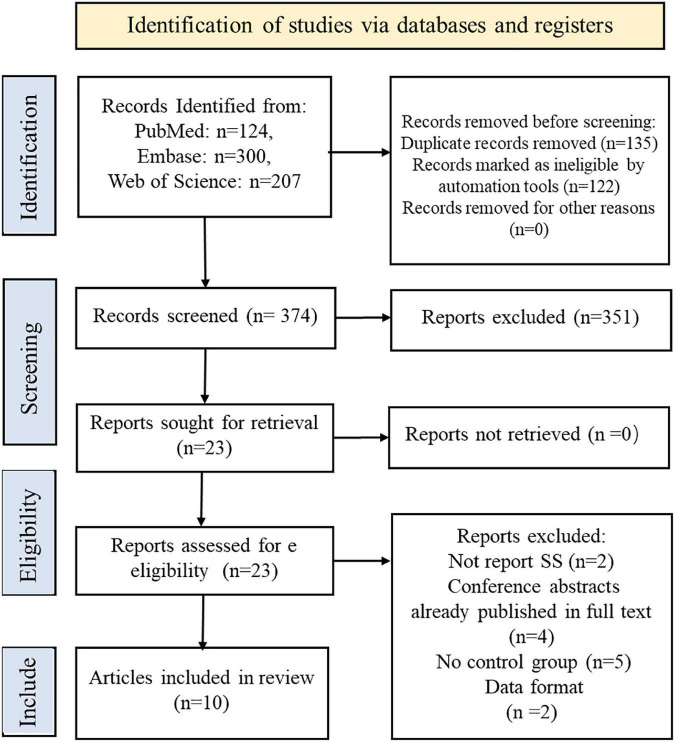
Flow diagram of the study selection process.

The screening process is summarized in the flow chart (Figure 1). We identified 631 records from PubMed, Embase, Web of Science databases according to a preformulated search strategy. After reviewing titles and abstracts, we excluded 135 studies with duplication and 122 irrelevant studies. The remaining 351 excluded studies were reviews, systematic reviews, animal experiments, and conference abstracts. After further reviewing the full text, 13 studies were excluded due to a lack of control groups or incomplete data. Ultimately, 10 studies were included in this meta-analysis (Wu et al., 2017; Chang et al., 2018; Chen et al., 2018, 2019; Liliang et al., 2018; Lin et al., 2018; Hou et al., 2019; Ju et al., 2019; Hsu et al., 2020; Park et al., 2021).

### Study characteristics

All included studies were published between 2017 and 2021. Of the 10 included studies, three were case-control studies (Wu et al., 2017; Chen et al., 2018; Park et al., 2021) and seven were cohort studies (Chang et al., 2018; Liliang et al., 2018; Lin et al., 2018; Chen et al., 2019; Hou et al., 2019; Ju et al., 2019; Hsu et al., 2020). One was conducted in South Korea (Park et al., 2021), and nine reported the scenarios in China (Wu et al., 2017; Chang et al., 2018; Chen et al., 2018, 2019; Liliang et al., 2018; Lin et al., 2018; Hou et al., 2019; Ju et al., 2019; Hsu et al., 2020). Participant sample sizes ranged from 1,929 to 85,342, with an overall mean age from 45 to 63.7 years. Participants diagnosed with Alzheimer’s disease, dementia, or PD at baseline were not included in any of the cohort studies or randomized controlled trials. Six of the 10 studies (Wu et al., 2017; Liliang et al., 2018; Chen et al., 2019; Hou et al., 2019; Ju et al., 2019; Hsu et al., 2020) were explicitly designed to assess the association between SS and dementia or PD. In comparison, in the remaining four studies (Chang et al., 2018; Chen et al., 2018; Lin et al., 2018; Park et al., 2021), SS was only part of their analysis to assess the relationship between autoimmune disease and dementia/PD. The definitions of dementia and PD varied between studies. Nine Taiwan, China-based studies used the ICD-9 standard, developed by neurologists based on the (UK) Parkinson’s Disease (PD) Society Brain Bank Clinical Diagnostic Criteria (Higgins et al., 2003; Stang, 2010; Moher et al., 2015; Wu et al., 2017; Chang et al., 2018; Chen et al., 2018, 2019; Liliang et al., 2018; Lin et al., 2018; Hou et al., 2019; Ju et al., 2019; Hsu et al., 2020), and one Korean study followed the ICD-10 classification standard (Park et al., 2021). All eligible studies were assessing dementia, PD, or SS as an outcome event. Seven studies specified the duration of follow-up, ranging from 5.18 to 15 years (Higgins et al., 2003; Stang, 2010; Moher et al., 2015; Wu et al., 2017; Chang et al., 2018; Liliang et al., 2018; Lin et al., 2018; Hou et al., 2019; Ju et al., 2019; Hsu et al., 2020). One study mentioned the follow-up but did not specify the duration (Chen et al., 2019), and the follow-up duration was missed in two studies (Chen et al., 2018; Park et al., 2021). The main characteristics of these studies are summarized in Table 1.

**TABLE 1:** The characteristics of included studies.

Study, year	Design	Country	Sources of participants	No. (expose/unexposed)	Occupation	Mean age years (SS/ control) (years)	Identification of SS cohort	Control group	Follow up (SS/control) (years)	Diagnosis of dementia/PD	Exposure duration (mean, years)	HR	95%LL	95%UL	Adjustment variables	Subgroup analysis
Park et al. (2021)	case- control, retrospective	Korean	NHIS-NSC database	589 (108/481)	Not reported	Not reported	SS: ICD-10 codes (M35)	Sex and age-matched, randomly selected from the original population	Not reported	prescribed With acetylcholinesterase inhibitors (AChEIs) at least once along with the symptoms outlined in the International Classification of Diseases (ICD)-10 code	Not reported	1.1	0.88	1.38	Age, sex, income, residence, city size, comorbidities	Not reported
Chen et al. (2018)	case-control, retrospective	China	Longitudinal health insurance database	69869 (9918/59951)	Not reported	Not reported/73.1 ± 9	SS: ICD-9-CM codes 710.2	Sex and index date with a match ratio 1 : 6, randomly selected from the original population	Not reported	Diagnosis and code (ICD-9-CM codes 290.0–290.4, 294.1, 331.0)	Not reported	1.28	1.11	1.47	Sex, age, and comorbidities	Gender, age
Wu et al. (2017)	case-control, retrospective	China	Longitudinal health insurance database	1,929 (1,036/893)	Not reported	63.7 ± 9.4/63.6 ± 9.3	SS: ICD-9-CM codes 710.2	Sex, age, and index date matched, randomly selected from the original population	≥9 years	Diagnosis and code (ICD-9-CM 332)	Not reported	1.38	1.15	1.66	Not reported	Not reported
Chen et al. (2019)	Cohort, retrospective	China	Longitudinal health insurance database	8126 (4063/4063)	Not reported	63.7 ± 9.4/63.6 ± 9.3	SS: ICD-9-CM codes 710.2	Sex and age-matched, randomly selected from the original population	12	Diagnosis and code (ICD-9-CM)	Not reported	1.21	1.02	1.45	Age, gender, hypertension, hyperlipidemia, Parkinson’s disease, and insomnia	Age, gender
Ju et al. (2019)	Cohort, retrospective	China	Taiwan’s national health insurance research database	63,200 (12,640/50,560)	Not reported	mean: 54	SS: ICD-9-CM codes 710.2	Sex and age-matched, randomly selected from the original population	5.21 ± 3.15/ 5.18±3.16	Diagnosis and code (ICD-9-CM 332)	Not reported	1.23	1.16	1.3	Age, and comorbidities of diabetes, hypertension, hyperlipidemia, coronary artery disease, head injury, depression, stroke	Age, sex, follow time
Liliang et al., 2018	Cohort, retrospective	China	Taiwan’s national health insurance research database	26,778 (4,463/22,315)	Not reported	Not reported	SS: ICD-9-CM codes 710.2	Sex and age-matched, randomly selected from the original population	10	Diagnosis and code (ICD-9-CM codes 331.0)	Not reported	2.69	1.07	6.76	Age, sex, and the geographical region, diabetes, hyperlipidemia, hypertension, coronary artery disease, heart failure, atrial, fibrillation, and stroke	Age
Hou et al. (2019)	Cohort, retrospective	China	Taiwan’s national health insurance research database	85,342 (17,072/68,270)	Not reported	54.20/54.02	SS: ICD-9-CM codes 710.2	Sex and age-matched, randomly selected from the original population	Not reported	Diagnosis and code (ICD-9-CM: 331.0, 290.0–290.3, 290.4, 294.1, 331.1–331.2, 331.82)	Not reported	1.246	1.123	1.384	Age, gender, comorbidities	Gender, age
Hsu et al. (2020)	Cohort, retrospective	China	Taiwan’s national health insurance research database	85,122 (17,028/68,094)	Not reported	Not reported	SS: ICD-9-CM codes 710.2	Sex and age-matched, randomly selected from the original population	15	Diagnosis and code (ICD-9-CM 332)	Not reported	1.23	1.07	1.42	Age, gender, comorbidities	Age, gender
Chang et al. (2018)	Cohort, retrospective	China	Taiwan’s national health insurance research database	11846 (8422/138424)	Not reported	≥45	SS:ICD-9-CM codes 710.2	Randomly selected from the original population	6.00 ± 5.71/6.40 ± 6.21	Diagnosis and code (ICD-9-CM 332)	Not reported	1.56	1.35	1.79	Age, sex, comorbidities	Age
Lin et al. (2018)	Cohort, retrospective	China	Taiwan’s national health insurance research database	8,449 (408/8,041)	Not reported	≥45	SS:ICD-9-CM codes 710.2	Sex and age-matched, randomly selected from the original population	5.97 ± 3.05/6.37 ± 2.95	Diagnosis and code (ICD-9-CM 332)	Not reported	1.46	1.32	1.63	Age, sex, comorbidities	Age

The Newcastle-Ottawa Quality Assessment Scale scores for the included studies are shown in Table 2. All 10 included studies were considered as high-quality; all of them scored 9, except for the study by Hou et al. (2019), which was rated as 8 because the specific follow-up time was not reported.

**TABLE 2:** Quality assessment of included studies.

Study (cohort)	Representativeness of exposed cohort	Selection of non-exposed cohort	Ascertainment of exposure	Outcome not present before study	Comparability	Assessment of outcome	Follow-up long enough	Adequacy of follow up	Quality score
Chen et al. (2018)	*	*	*	*	**	*	*	*	9
Ju et al. (2019)	*	*	*	*	**	*	*	*	9
Liliang et al. (2018)	*	*	*	*	**	*	*	*	9
Hou et al. (2019)	*	*	*	*	**	*		*	8
Hsu et al. (2020)	*	*	*	*	**	*	*	*	9
Chang et al. (2018)	*	*	*	*	**	*	*	*	9
Lin et al. (2018)	*	*	*	*	**	*	*	*	9

**Study (case-control)**	**Case definition**	**Representativeness of the cases**	**Selection of Controls**	**Definition of Controls**	**Comparability**	**Ascertainment of exposure**	**Same method**	**Non-response rate**	**Quality score**

Park et al. (2021)	*	*	*	*	**	*	*	*	9
Chen et al. (2019)	*	*	*	*	**	*	*	*	9
Wu et al. (2017)	*	*	*	*	**	*	*	*	9

Follow-up long enough: *Median/mean follow-up of more than 5 years or maximum follow-up of more than 10 years was considered enough.

Adequacy of follow up: *A follow-up rate of > 80% and a descriptive analysis of those who were missed was considered adequate.

**Represents a score.

### Association between Sjögren’s syndrome and dementia

**FIGURE 2:**
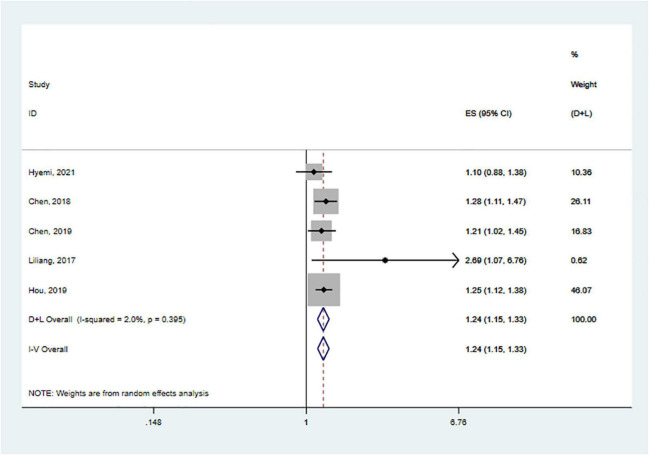
Forest plot of the association between SS and risks of dementia.

We performed meta-analyses and calculated pooled effect estimates for five studies that included 190 704 subjects (Chen et al., 2018, 2019; Liliang et al., 2018; Hou et al., 2019; Park et al., 2021). Heterogeneity between included studies was subtle [*I*^2^ = 2.0%, p _(heterogeneity)_ = 0.395]. Therefore, a fixed-effects model was used to pool the effect size of each study to determine the association between SS and the risk of dementia (Figure 2). The overall pooled results showed that SS was associated with an increased risk of dementia (HR = 1.24, 95% CI: 1.15–1.33, *p* < 0.001).

Our subgroup analyses were conducted by age (<65 and >65) and sex (male and female). Corresponding subgroup analyses of follow-up time and comorbidities on risk were not performed due to the lack of data on the effect (Table 3).

**TABLE 3:** Subgroup analysis of the association between SS and risk of dementia.

Subgroup	No. of studies	*I* ^2^	P _heterogeneity_	Hazard ratio	95% CI	Pooled model	*P*(overall effect)
**Age**							
<65	2	0.0	0.518	1.53	1.19–1.98	Fixed-effects model	0.001
>65	3	49.1	0.140	1.26	1.14–1.39	Fixed-effects model	0.000
**Gender**							
Female	2	0.0	0.889	1.25	1.13–1.38	Fixed-effects model	0.000
Male	2	0.0	0.740	1.29	1.08–1.55	Fixed-effects model	0.005

*N*, number; HR, hazard ratio.

In terms of stratified analysis for age, people with SS had a 26% higher relative risk of dementia than those without SS among those over 65 (HR = 1.26; 95% CI: 1.14–1.39; *p* = 0.004), and this risk was similar in patients with SS under 65 years of age (HR = 1.53, 95% CI: 1.19–1.98; *p* < 0.001). Two studies were included in the subgroup analysis by sex (Chen et al., 2018; Hou et al., 2019). Females with SS had a similar risk of developing PD as males. (Females: HR = 1.25; 95% CI: 1.13–1.38; *p* < 0.001 vs. Male: HR = 1.29; 95% CI: 1.08–1.55; *p* = 0.005, respectively).

### Evaluation for publication bias and sensitivity analysis

**FIGURE 3:**
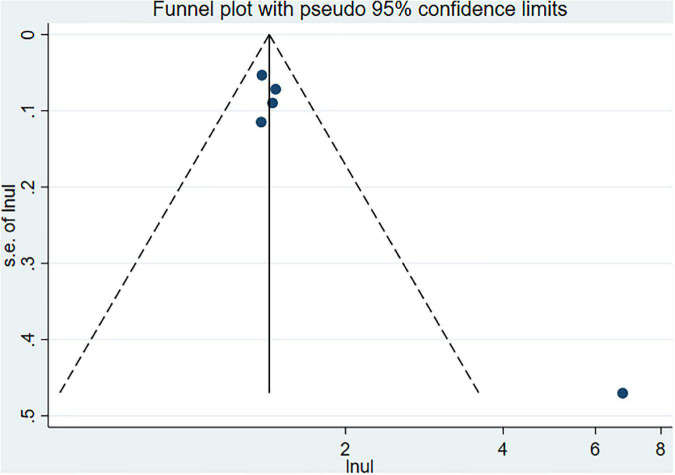
The funnel plot for dementia.

**FIGURE 4:**
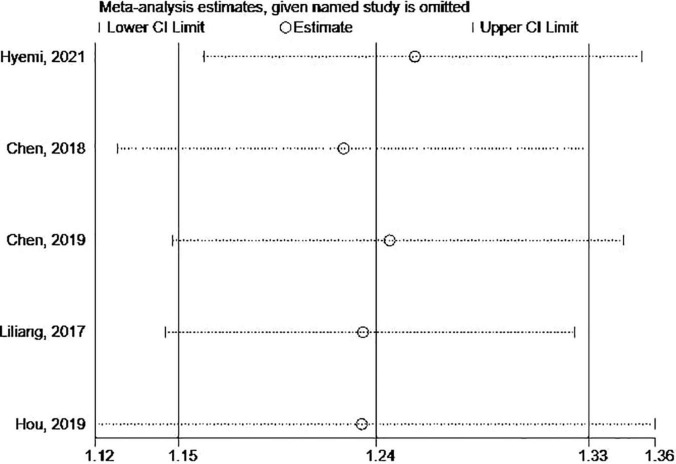
The sensitivity analysis of the association between SS and risks of dementia.

The Begg’s and Egger’s tests and funnel plot showed no publication bias in the current studies (Begg = 1.000, Egger = 0.446) (Figure 3). Furthermore, sensitivity analysis of the pooled results showed that individual studies did not substantially affect the association between SS and dementia (Figure 4).

### Association between Sjögren’s syndrome and Parkinson’s disease

**FIGURE 5:**
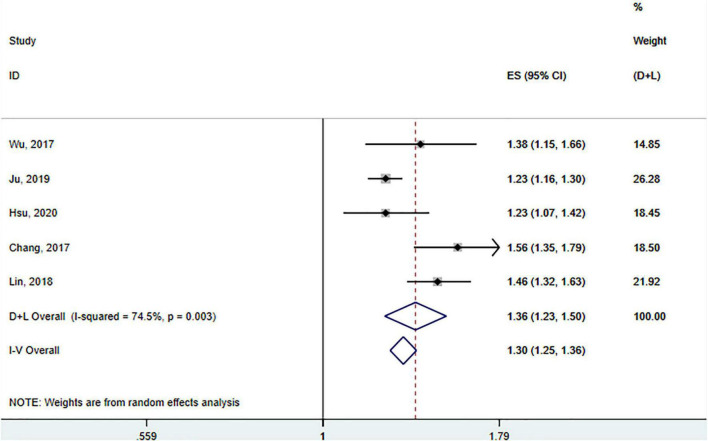
Forest plot of the association between SS and risks of PD.

A total of five studies involving 163 522 participants (Wu et al., 2017; Chang et al., 2018; Lin et al., 2018; Ju et al., 2019; Hsu et al., 2020) assessed the association between SS and the risk of PD. Tests for heterogeneity showed large heterogeneity between studies (*I*^2^ = 74.5%, p _(heterogeneity)_ = 0.003), so a random-effects model was adopted. Pooled results showed that SS was significantly associated with a subsequent increased risk of PD (HR = 1.36; 95% CI: 1.23–1.50, *p* < 0.001) (Figure 5).

We conducted a meta-analysis of subgroups according to age, sex, and duration of follow-up (Table 4). In the subgroup analysis based on age, the risk of developing PD was 1.56 and 1.31 times higher in SS patients under and over 65 than in non-SS patients (Chang et al., 2018; Lin et al., 2018; Ju et al., 2019; Hsu et al., 2020), respectively (<65: HR = 1.56; 95% CI: 1.37–1.78 vs. > 65: HR = 1.31; 95% CI: 1.14–1.51, respectively), indicating that age had no significant effect on the increased risk.

**TABLE 4:** Subgroup analysis of the association between SS and risk of PD.

Subgroup	No. of studies	*I* ^2^	P _heterogeneity_	Hazard ratio	95% CI	Pooled model	*P*
**Age**							
<65	4	0.0	0.398	1.56	1.37–1.78	Fixed-effects model	0.000
>65	4	65.5	0.034	1.31	1.14–1.51	Random-effects model	0.000
**Gender**							
Female	2	0.0	1.000	1.28	1.21–1.35	Fixed-effects model	0.000
Male	2	0.0	0.920	1.00	0.87–1.16	Fixed-effects model	0.962
**Follow time, years**							
<3	1	NA	NA	1.25	1.18–1.32	NA	0.000
4–6	1	NA	NA	1.22	1.14–1.31	NA	0.000
7–9	1	NA	NA	1.26	1.12–1.42	NA	0.000
≥9	1	NA	NA	1.93	1.66–2.23	NA	0.000

*N*, number; HR, hazard ratio.

In the subgroup analysis by sex, two cohort studies (Ju et al., 2019; Hsu et al., 2020) examined the risk of subsequent PD in male and female SS patients. The pooled HR for PD was 1.28 (95% CI: 1.21–1.35; *p* = 0.000) in female SS patients and 1.00 (95% CI: 0.87–1.16, *p* = 0.962) in male SS patients. These results revealed that the correlation between SS and PD risk seemed to be applicable primarily to female patients.

Only Park et al. (2021) assessed the association between the duration of follow-up on SS and subsequent PD risk. The result showed that the association between SS and PD risk was higher for long-term follow-up (≥9 years) than for follow-up periods of less than 9 years.

### Evaluation for publication bias and sensitivity analysis

**FIGURE 6:**
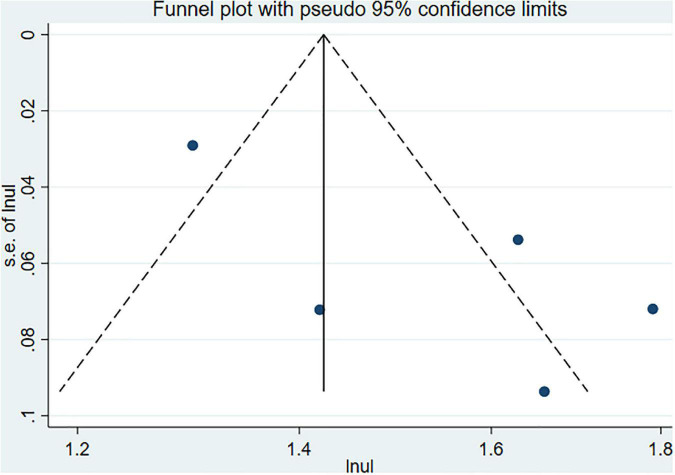
The funnel plot for PD.

**FIGURE 7:**
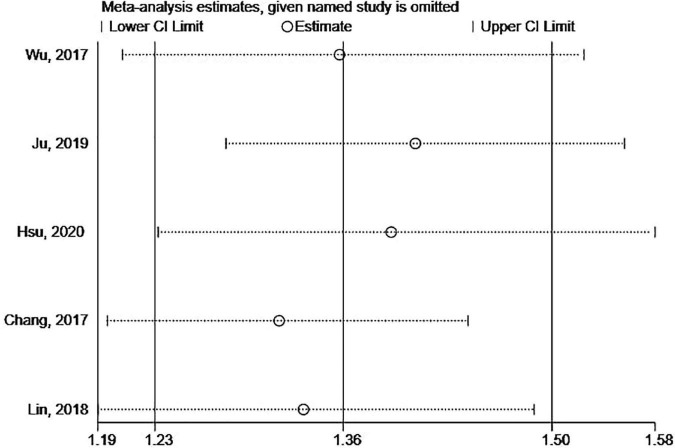
The sensitivity analysis of the association between SS and risks of PD.

Begg’s and Egger’s tests as well as funnel plots were used to assess potential publication bias. No potential publication bias was present (Begg = 0.806 and Egger = 0.242) (Figure 6). The robustness of the results was assessed by deleting each study in turn. The exclusion of any individual study did not affect the conclusions (Figure 7).

## Discussion

### Main findings

To our knowledge, this meta-analysis is the first to comprehensively investigate and quantify the association between SS and the risk of dementia/PD. We found a 1.24-fold greater risk of dementia in people with SS compared to the general population; age and gender appear to have no significant effect on this risk. The risk of PD in SS patients was 1.36 times higher in SS individuals. This elevated risk, interestingly, appeared to be prevalent solely in female individuals. Age had no significant effect on the risk of developing PD in SS patients. Based on the fact that over 10,000 patients with both SS and dementia or both SS and PD were involved, the study provided convincing information for the association between SS and PD.

### Influence of Sjögren’s syndrome on dementia

In recent years, the prevalence of dementia has been rising at an alarming rate in our aging population (Raz et al., 2016). Dementia mainly includes Alzheimer’s disease, Lewy body dementia, frontotemporal dementia, vascular dementia, and mixed dementia (Marson et al., 2021). It is estimated that 46.8 million people worldwide were living with dementia in 2015. This number is expected to reach 131.5 million by 2050 (Timoszuk et al., 2018). Amyloid-beta (Aβ) peptides and neuroinflammation are the most notable indicators of the disease (Leng and Edison, 2021). Although the molecular mechanisms of tissue damage in these dementias are yet to be fully understood, neuroinflammation and other specific changes in these neurodegenerative diseases, have become the focus of new research (Hensley, 2010; Calsolaro and Edison, 2016). The risk of PD as a comorbidity in patients with SS, one of the most common inflammatory rheumatic diseases with a prevalence between 1:100 and 1:1,000 (Witte, 2019), has received increasing attention (Liliang et al., 2018; Chen et al., 2019; Hou et al., 2019). Since the higher risk of dementia in SS patients may produce significant public health consequences, it is necessary to explore the possibility of SS as an early manifestation of dementia and obtain the potential benefits of early treatment with immunomodulatory or immunosuppressive drugs at an early stage.

The influence of neuroinflammation on dementia remains a controversial topic. The interaction between SS and dementia is becoming more obvious, yet the factors that contribute to progression are unclear. Vasculitis, autoantibodies, immune complex deposition, and cellular inflammation are potential pathways through which SS may elevate the risk of dementia, resulting in nerve damage, cognitive impairment, and initial dementia (Tobón et al., 2012). Previous studies have suggested that inflammation may be a significant event in the pathophysiology of dementia (Newcombe et al., 2018; Webers et al., 2020). Recent studies have found that SS can produce local and systemic inflammation, which in turn leads to a large elevation in IL-1β and TNF-α in vivo (Lisi et al., 2011, 2012) and directly or indirectly causes neuronal damage, followed by AD (Ye et al., 2013). Furthermore, Choi et al. (2018) observed that damage to hippocampal neurogenesis in the early stages of AD may increase the vulnerability of hippocampal neurons, resulting in more severe cognitive impairment and neuronal loss in the later stages of AD. Animal experiments by Vom Berg demonstrated that the natural antibody ustekinumab inhibited the pro-inflammatory cytokines IL-12 and IL-23, which are associated with the accumulation of amyloid, and improved cognitive performance in mice (Sue and Griffin, 2013). Although there is no direct evidence on the occurrence of neuroinflammation in the human AD brain, the findings above suggest that neuroinflammation and altered neurogenesis are linked in AD models. However, further studies are needed to clarify the exact causal relationship between these two phenomena.

Noteworthily, Hou et al. (2019) observed that SS patients with a combination of any other comorbidities (diabetes, hypertension, cardiovascular disease, stroke, and severe mental illnesses) had a higher risk of dementia than non-SS patients without comorbidities. This is consistent with the findings of Chen et al. (2019) who found that SS patients with co-occurring hypertension, PD, and insomnia were more likely to develop dementia. Some studies have confirmed that SS patients are more likely to develop hyperlipidemia, hypertension, and other disorders (Ramos-Casals et al., 2007; Pérez-De-Lis et al., 2010). Many dementias, on the other hand, are associated with diabetes, metabolic syndrome, and cardiovascular disease, suggesting that underlying disease factors may skew the association between SS and increased risk of dementia. This may explain why patients with SS who have comorbidities are more likely to develop dementia. Furthermore, there is no association between substantial cognitive impairment (including dementia) and the use of cardiovascular or hypertensive medications in people with these comorbidities (Hou et al., 2019).

### Influence of Sjögren’s syndrome on Parkinson’s disease

PD is the second most common neurodegenerative disease after Alzheimer’s disease, and it is expected to place an increasing medical and economic burden on society as the population ages (de Lau and Breteler, 2006). Typical PD symptoms are resting tremor, rigidity, bradykinesia, postural instability (Aarsland et al., 2017), and pathology characterized by degeneration and death of dopaminergic neurons in the substantia nigra (Obeso et al., 2008). Visser et al. (1993) first reported a case of hemiparkinsonism (partial Parkinson’s syndrome) in a patient who presented with both signs and symptoms of the primary SS in 1993. Mochizuki et al. (1997) discovered a man diagnosed with Parkinson’s syndrome who also had SS. His PD was believed to be caused by SS. More recently, Kchaou et al. (2015) concluded that neuroinflammatory processes appear to exist between two completely different diseases, PD and pSS, based on observations of pathophysiological findings in PD patients. A growing number of researchers recognized the risk of PD as a comorbidity in SS patients, and several immune system-mediated mechanisms have been proposed to account for the possible pathogenic mechanism of autoantibodies inducing dopaminergic cell death (Kunas et al., 1995; Monahan et al., 2015). SS and other autoimmune diseases form a spectrum of systemic symptoms ranging from organ-specific to multi-organ involvement. Patients suffer from a significant symptom-related burden, followed by a drop in health-related quality of life and productivity (Meijer et al., 2009). Therefore, health education and risk factor modification are essential for this patient group.

It is crucial to determine the causal relationship between SS and PD. In several of the included studies, all patients with PD were diagnosed after an episode of SS (Wu et al., 2017; Chang et al., 2018; Lin et al., 2018; Ju et al., 2019; Hsu et al., 2020). In the study by Ju et al. (2019), there was a 93% greater risk of PD with SS when the follow-up duration was longer than 9 years compared to subjects without SS. As a result, the available evidence suggests that SS is a risk factor for PD rather than a shared risk factor or a factor with an inverse correlation.

SS is known to be mediated by the interaction of genetic, epigenetic, and environmental factors causing immune dysregulation, which leads to an aggressive autoimmune disease affecting the central nervous system (CNS) and peripheral nervous system (Morgen et al., 2004), often exhibiting a higher female bias (Chatzis et al., 2021). Our study also found that the correlation between SS and PD risk appears to occur primarily in female patients, as opposed to male SS patients, which may be explained by that higher urate levels in men diminish the risk of PD, whereas this is not the case in women (Ascherio et al., 2009; O’Reilly et al., 2010). Autopsy analyses and experimental animal studies of human PD patients have shown that increased pro-inflammatory factors in the brain lead to neuronal degeneration and the development of PD (Wang et al., 2015). Furthermore, both anticardiolipin and anti-β2-glycoprotein-I levels have been reported to be higher in SS patients who develop PD (Mochizuki et al., 1997; Hassin-Baer et al., 2007). Inflammation may thus be a critical factor in the development of PD in patients with SS, yet neuroinflammation has been a controversial topic in the pathogenesis of PD (Figueiredo-Pereira et al., 2015; Renaud et al., 2015). A recent review explored the evidence for autoimmune involvement in PD and proposed targeted inflammatory therapy as a novel neuroprotective approach (Moehle and West, 2015).

Ju et al. (2019) explored the effect of immunosuppression on the risk of PD in patients with autoimmune diseases, including SS, and found that the risk of PD was significantly higher in participants with SS who received hydroxychloroquine, compared to those without SS (HR = 1.46, 95% CI: 1.34–1.59), but the risk of PD in SS participants receiving non-hydroxychloroquine immunosuppressive therapy was relatively low (HR = 0.86; 95% CI: 0.73–1.01). It is, therefore, justifiable to assume that non-hydroxychloroquine immunosuppression plays a crucial role in lowering the risk of PD. The neuroprotective potential of hydroxychloroquine has been debated, and its efficacy in treating systemic lupus erythematosus (Ruiz-Irastorza et al., 2010) and rheumatoid arthritis (Suarez-Almazor et al., 2000) has been widely recognized. Still, its efficacy in the treatment of SS remains unclear. In a study by Fox et al. (1996) on hydroxychloroquine in the treatment of SS, regular use of hydroxychloroquine was found to be effective in treating SS, providing relief from fatigue and arthralgia. However, several studies have indicated that taking hydroxychloroquine for SS had no therapeutic effect for people with pSS when compared to placebo treatment (Kruize et al., 1996). Immunotherapy-related neurological disorders have been progressively documented in recent years (Villoslada et al., 2008; Prior, 2015; General-López, 2018). Future research should concentrate on the association between autoimmune disease and PD, as well as the relation between immunosuppression and PD.

### Limitations

There are still some limitations to our meta-analysis. First, we could not distinguish whether SS was primary or secondary to another autoimmune disease in our meta-analysis. Failure to fully elucidate the clinical severity of SS and the subtypes of dementia and PD may lead to inaccurate estimates of their genuine association. Secondly, most of the eligible studies were conducted in Taiwan region (China), which may lead to geographical bias and relatively high overlap of included samples. Therefore, it is unclear whether this association holds for other races and regions in the world. In addition, none of the 10 included studies assessed the effect of monitoring bias on outcomes. Since subjects with SS typically experienced more medical visits than those without SS, they were more likely to be diagnosed with dementia or PD. In this way, the risk of dementia or PD for subjects with SS may be skewed higher in the overall analysis results. Finally, all original studies were designed retrospectively and their data collection did not consider the need for specific scientific research, thus limiting the completeness and homogeneity of the data.

## Conclusion

The results of this study reveal that patients with SS are at significantly elevated risk of developing PD and dementia and that regular neurological screening of SS patients may be warranted. More prospective studies from different regions are necessary to elucidate the underlying mechanisms and to further characterize the impact of SS on development of PD and dementia.

## Data availability statement

The original contributions presented in this study are included in the article/Supplementary material, further inquiries can be directed to the corresponding author/s.

## Author contributions

Z-ZW, M-SL, and ZS developed the protocol, participated in the literature search, extracted data, and drafted the manuscript. X-LZ and M-LZ was responsible for the analysis and interpretation of the data. KX contributed to statistical expertise. FZ supervised the study. All authors contributed to the article and approved the submitted version.
