# Supplementary material for: Risk of dementia or Parkinson’s disease in the presence of Sjögren’s syndrome: A systematic review and meta-analysis
Source: Front Integr Neurosci. 2022 Nov 7;16:1027044. doi: 10.3389/fnint.2022.1027044 (PMC9676366; doi:10.3389/fnint.2022.1027044)
Supplement: Supplementary file 1 [file Data_Sheet_1.docx]

**PubMed**

((("Parkinson Disease"[Mesh]) or Idiopathic Parkinson's Disease or Lewy Body Parkinson's Disease or Parkinson's Disease, Idiopathic or Parkinson's Disease, Lewy Body or Parkinson Disease, Idiopathic or Parkinson's Disease or Idiopathic Parkinson Disease or Lewy Body Parkinson Disease or Primary Parkinsonism or Parkinsonism, Primary or Paralysis Agitans) OR (("Dementia"[Mesh]) or Dementias or Amentia or Amentias or Senile Paranoid Dementia or Dementias, Senile Paranoid or Paranoid Dementia, Senile or Paranoid Dementias, Senile or Senile Paranoid Dementias or Familial Dementia or Dementia, Familial or Dementias, Familial or Familial Dementias) or (("Alzheimer Disease"[Mesh]) or Alzheimer Dementia or Alzheimer Dementias or Dementia, Alzheimer or Alzheimer's Disease or Dementia, Senile or Senile Dementia or Dementia, Alzheimer Type or Alzheimer Type Dementia or Alzheimer-Type Dementia (ATD) or Alzheimer Type Dementia (ATD) or Dementia, Alzheimer-Type (ATD) or Alzheimer Type Senile Dementia or Primary Senile Degenerative Dementia or Dementia, Primary Senile Degenerative or Alzheimer Sclerosis or Sclerosis, Alzheimer or Alzheimer Syndrome or Alzheimer's Diseases or Alzheimer Diseases or Alzheimers Diseases or Senile Dementia, Alzheimer Type or Acute Confusional Senile Dementia or Senile Dementia, Acute Confusional or Dementia, Presenile or Presenile Dementia or Alzheimer Disease, Late Onset or Late Onset Alzheimer Disease or Alzheimer's Disease, Focal Onset or Focal Onset Alzheimer's Disease or Familial Alzheimer Disease (FAD) or Alzheimer Disease, Familial (FAD) or Familial Alzheimer Diseases (FAD) or Alzheimer Disease, Early Onset or Early Onset Alzheimer Disease or Presenile Alzheimer Dementia)) AND (("Sjögren's syndrome"[Mesh]) or Sjogrens Syndrome or Syndrome, Sjogren's or Sjogren Syndrome or Sicca Syndrome or Syndrome, Sicca)))

**web of science**

(((Parkinson or Parkinson Disease or Lewy Body Parkinson's Disease or Parkinson's Disease, Idiopathic or Parkinson's Disease, Lewy Body or Parkinson Disease, Idiopathic or Parkinson's Disease or Idiopathic Parkinson Disease or Lewy Body Parkinson Disease or Primary Parkinsonism or Parkinsonism, Primary or Paralysis Agitans) or (dementia or Dementias or Amentia or Amentias or Senile Paranoid Dementia or Dementias, Senile Paranoid or Paranoid Dementia, Senile or Paranoid Dementias, Senile or Senile Paranoid Dementias or Familial Dementia or Dementia, Familial or Dementias, Familial or Familial Dementias) or (Alzheimer or Alzheimer Dementia or Alzheimer Dementias or Dementia, Alzheimer or Alzheimer's Disease or Dementia, Senile or Senile Dementia or Dementia, Alzheimer Type or Alzheimer Type Dementia or Alzheimer-Type Dementia (ATD) or Alzheimer Type Dementia (ATD) or Dementia, Alzheimer-Type (ATD) or Alzheimer Type Senile Dementia or Primary Senile Degenerative Dementia or Dementia, Primary Senile Degenerative or Alzheimer Sclerosis or Sclerosis, Alzheimer or Alzheimer Syndrome or Alzheimer's Diseases or Alzheimer Diseases or Alzheimers Diseases or Senile Dementia, Alzheimer Type or Acute Confusional Senile Dementia or Senile Dementia, Acute Confusional or Dementia, Presenile or Presenile Dementia or Alzheimer Disease, Late Onset or Late Onset Alzheimer Disease or Alzheimer's Disease, Focal Onset or Focal Onset Alzheimer's Disease or Familial Alzheimer Disease (FAD) or Alzheimer Disease, Familial (FAD) or Familial Alzheimer Diseases (FAD) or Alzheimer Disease, Early Onset or Early Onset Alzheimer Disease or Presenile Alzheimer Dementia)) AND ((Sjogren's syndrome or Sjogrens Syndrome or Syndrome, Sjogren's or Sjogren Syndrome or Sicca Syndrome or Syndrome, Sicca))

**embase**

Sjogren's syndrome

'dacryosialoadenopathia atrophicans':ab,ti OR 'dyssecretosis, mucoserous':ab,ti OR 'gougerot houwer sjoegren syndrome':ab,ti OR 'gougerot mulock houwer sjoegren syndrome':ab,ti OR 'gougerot sjoegren disease':ab,ti OR 'gougerot sjoegren syndrome':ab,ti OR 'gougerot sjogren disease':ab,ti OR 'gougerot sjogren syndrome':ab,ti OR 'gougerot-sjogren syndrome':ab,ti OR 'mikulicz gougerot sjoegren syndrome':ab,ti OR 'mikulicz radecki syndrome':ab,ti OR 'mucoserous dyssecretosis':ab,ti OR 'mukilicz radecki syndrome':ab,ti OR 'oculobuccopharyngeal dryness':ab,ti OR 'rheumatic sialosis; sialosis, rheumatic':ab,ti OR 'sicca syndrome; sjoegren disease':ab,ti OR 'sjogren disease':ab,ti OR 'sjogren syndrome':ab,ti OR 'sjogrens syndrome'

#1 OR #2

'parkinson disease'/exp

'idiopathic parkinsonism':ab,ti OR 'lewy bodies of parkinson disease':ab,ti OR 'lewy bodies of parkinsons disease':ab,ti OR 'lewy body parkinson disease':ab,ti OR 'lewy body parkinsons disease':ab,ti OR 'paralysis agitans':ab,ti OR 'parkinson dementia complex':ab,ti OR 'parkinsons disease':ab,ti OR 'primary parkinsonism'

#4 OR #5

'dementia'/exp

'amentia':ab,ti OR 'demention'

#7 OR #8

'alzheimer disease'/exp

'alzeimer disease':ab,ti OR 'alzeimers disease':ab,ti OR 'alzheimer dementia':ab,ti OR 'alzheimer fibrillary change':ab,ti OR 'alzheimer fibrillary lesion':ab,ti OR 'alzheimer neurofibrillary change':ab,ti OR 'alzheimer neurofibrillary degeneration':ab,ti OR 'alzheimer neuron degeneration':ab,ti OR 'alzheimer perusini disease':ab,ti OR 'alzheimer sclerosis':ab,ti OR 'alzheimer syndrome':ab,ti OR 'alzheimers disease':ab,ti OR 'cortical sclerosis, diffuse':ab,ti OR 'dementia, alzheimer':ab,ti OR 'diffuse cortical sclerosis':ab,ti OR 'late onset alzheimer disease'

#10 OR #11

#6 OR #9 OR #12

#3 AND #13
